# Immune-inflammatory biomarkers and the risk of cardiac injury in COVID-19 patients with diabetes: a retrospective cohort study

**DOI:** 10.1186/s12933-022-01625-2

**Published:** 2022-09-19

**Authors:** Yi Bo, Cai Yuli, Wang Ye, Li Junfeng, Chen Xiaolin, Bao Yan, Wen Zhongyuan

**Affiliations:** Department of Endocrinology, Renmin Hospital of Wuhan University, Wuhan, 430060 China

**Keywords:** Cardiac injury, COVID-19, Diabetes mellitus, Immune-inflammatory biomarkers, Mortality

## Abstract

**Background:**

To determine the risk-assessment role of the immune-inflammatory biomarkers on myocardial damage in COVID-19 patients with diabetes mellitus (DM).

**Methods:**

This retrospective study was conducted on 822 COVID-19 inpatients from 1 January to 10 March 2020 at Renmin Hospital of Wuhan University. The demographic data, clinical data, and immune-inflammatory parameters of participants were collected. The predictors of cardiac injury were assessed by Logistics regression analysis.

**Results:**

A total of 246 COVID-19 inpatients were diagnosed with DM (29.9%). The incidence of cardiac injury was higher in patients with DM than in non-DM cases (28.9% vs 9.0%, p < 0.001), even grouped by age, gender, and the level of fasting plasma glucose (FPG). The mortality in diabetic COVID-19 patients with cardiac injury and without cardiac injury was 42.9% and 3.4%, respectively (*p* < 0.001). COVID-19 patients with DM and cardiac injury presented a decreased number of immunocyte subsets, lower C3 concentration, and a higher level of interleukin-6 (IL-6) and immunoglobulin A (IgA). The independent risk factors for cardiac injury in COVID-19 patients with DM were CD3^+^CD4^+^ T cells counts ≤ 288 cells/μl (adjusted Odds ratio (OR), 2.501; 95% confidence interval (CI) 1.282–4.877; *p* = 0.007) and IL-6 > 25.68mpg/ml (adjusted OR, 4.345; 95% CI 2.192–10.374; *p* < 0.001) (all *P*_interaction_ < 0.05).

**Conclusions:**

For diabetic patients with COVID-19, cardiac injury not only induce severer immune-inflammatory responses, but also increase in-hospital mortality. The decreased number of CD3^+^CD4^+^ T cells and increased IL-6 are recommended to distinguish the people who refer to high risk of cardiac injury and mortality from those persons. However, it remains a testable theory whether decision-making strategies based on the risk status of cardiac injury in COVID-19 patients, especially with DM, would be expected to get better outcomes.

**Supplementary Information:**

The online version contains supplementary material available at 10.1186/s12933-022-01625-2.

## Background

The Coronavirus disease 2019 (COVID-19) pandemic caused by the severe acute respiratory syndrome coronavirus 2 (SARS-CoV-2) is a persistent serious challenge for global public health, even the use of coronavirus vaccines [1]. Our previous study found that diabetes mellitus (DM) confer approximately 20% of in-hospital mortality of COVID-19 patients [2]. Similar findings were also reported in England, Italy, France, and America [3–6]. Notably, among COVID-19 patients, the prevalence of cardiac injury in DM is nearly twice as high as people in non-DM [7, 8]. Importantly, higher cardiac troponin T (cTnI) levels, the biomarker of cardiac injury, were robustly associated with the severity and mortality of COVID-19 patients[5, 9–11] and adults with DM [12]. Thus, for guiding the effective clinical management of COVID-19 with DM, it is an urgent need to identify high-risk subgroups with elevated cardiac injury risk.

Dysregulation of the immune-inflammatory responses was the prominent characteristic of both COVID-19 patients with DM and with cardiac injury [2, 13–15]. These persons all present lymphopenia, T cell exhaustion, and elevated level of inflammatory factors, such as interleukin-6 (IL-6) [7, 14]. These changes have a dramatic relevance to the severity and mortality of COVID-19 [7, 14]. Inflammatory markers and lymphocytic infiltrates are also markedly elevated in the heart of deceased patients from cardiac pathological data [16, 17]. Notably, diabetes facilitates SARS-CoV-2 viral entry into the heart via the overexpression of cellular angiotensin-converting enzyme 2 (ACE2) and Transmembrane serine protease 2 (TMPRSS2) [18, 19]. Thus, immune-inflammatory responses may be a “bridge” between DM and myocardial injury in COVID-19. However, only one study of 124 COVID-19 patients reported that minimal lymphocyte percentage < 7.8% was an independent risk factor for cardiac injury [15]. Moreover, this study based on small sample sizes was not specifically designed for diabetic COVID-19 patients.

Herein, this retrospective study of 822 COVID-19 patients was conducted to comprehensively clarify the potential risk-assessment role of the immune-inflammatory reaction on cardiac injury in DM during hospitalization.

## Methods

### Study design

This retrospective study was conducted at Renmin Hospital of Wuhan University. All consecutive patients reviewed from 1 January to 10 March 2020 were regularly followed up to 26 April 2020, the day of the discharge of the last cases in Wuhan. Only confirmed COVID-19 patients were enrolled in the survey. The exclusion criterions included suspected cases, neonates, pregnancy, duplicated cases, and cases lack of cardiac biomarkers.

### Definitions

According to the 7th edition guideline published by the China National Health Commission (http://kjfy.meetingchina.org/msite/news/show/cn/3337.html), confirmed COVID-19 case was identified as positive for SARS-COV-2 after real-time reverse-transcriptase polymerase chain reaction (RT-PCR) test, high-throughput sequencing, and/or COVID-19 specific immunoglobulin M (IgM) and IgG antibodies examination. Cardiac injury was defined as the maximum level of cTnI > 99th percentile upper reference limit (URL) (0.04 ng/ml) after hospitalization, regardless of the new manifestations in echocardiography or electrocardiogram, based on previous clinical studies [9, 10, 13, 20]. DM was the person with a history of DM, and/or the use of antidiabetic therapies, and/or the presence of at least two abnormal blood glucose (fasting glucose ≥ 7.0 mmol/l and/or random glucose ≥ 11.1 mmol/l and/or hemoglobin A1c ≥ 6.5%). Coagulopathy was described as the score for sepsis-induced coagulopathy > 4.

### Data collection

We collected age, gender, initial symptoms, laboratory findings, history of comorbidities, treatments, records of chest computed tomographic (CT) scans, and clinical outcomes from the electronic medical records system by two independent investigators (Cai Yuli and Wang Ye).

The detection procedure of COVID-19 by PCR depended on sputum and throat swab samples was described in our previous study [2]. cTnI was evaluated by kit from Siemens based on the chemiluminescence immunotechnology. BD FACSCanto II Flow Cytometer was used to assess the proportions and numbers of total CD3^+^ T cells, CD3^+^CD4^+^ T cells, CD3^+^CD8^+^ T cells, CD16^+^CD56^+^ Natural Killer cells (NK cells), and CD19^+^ B cells subsets (BD Multitest). Serum levels of IgG, IgM, IgA, IgE, and complements component (C3, C4) were detected by rate nephelometry immunoassay (N Antiserum to Human Ig Kit series, Siemens, Germany). Cytometric Bead Array with the human helper T cells 1/2 cytokine kit II (BD Ltd., USA) was used to test the plasma levels of cytokines, including interleukin-2 (IL-2), IL-4, IL-5, IL-6, IL-10, tumor necrosis factor-α (TNF-α), and γ-interferon. All tests were conducted according to the manufacturer's instructions in routine clinical practices during the COVID-19 pandemic in our hospital.

### Statistical analyses

Categorical data and continuous data were presented as proportions (%) and median (interquartile range [IQR]) values, respectively. For continuous variables, we used the *t*-test or Mann–Whitney *U* test to compare the differences between COVID-19 patients with and without DM; otherwise, the one-way *ANOVA* or Kruskal–Wallis *H-*test was used depending on parametric or nonparametric data. Categorical variables were compared by the *χ*^2^ test or Fisher’s exact test. Survival curves were plotted using the Kaplan–Meier method, while the linear correlation was calculated by spearman’s correlation test. The cutoff value of immune-related biomarkers to differentiate between survivors and deceased were performed by the receiver operating characteristic curve (ROC). We also used Logistics regression analysis to determine the risk factors of cardiac injury and the interaction between DM and immune-related indicators.

All data were analyzed by SPSS Software V19.0 (IBM Corp.). Statistical charts were constructed using Prism 5 (GraphPad), Minitab Statistical Software V19 (Minitab LLC.), and MedCalc statistical software version 20.011 (MedCalc Software Ltd). A two-tailed *p*-value < 0.05 was considered statistically significant.

## Results

**Fig. 1:**
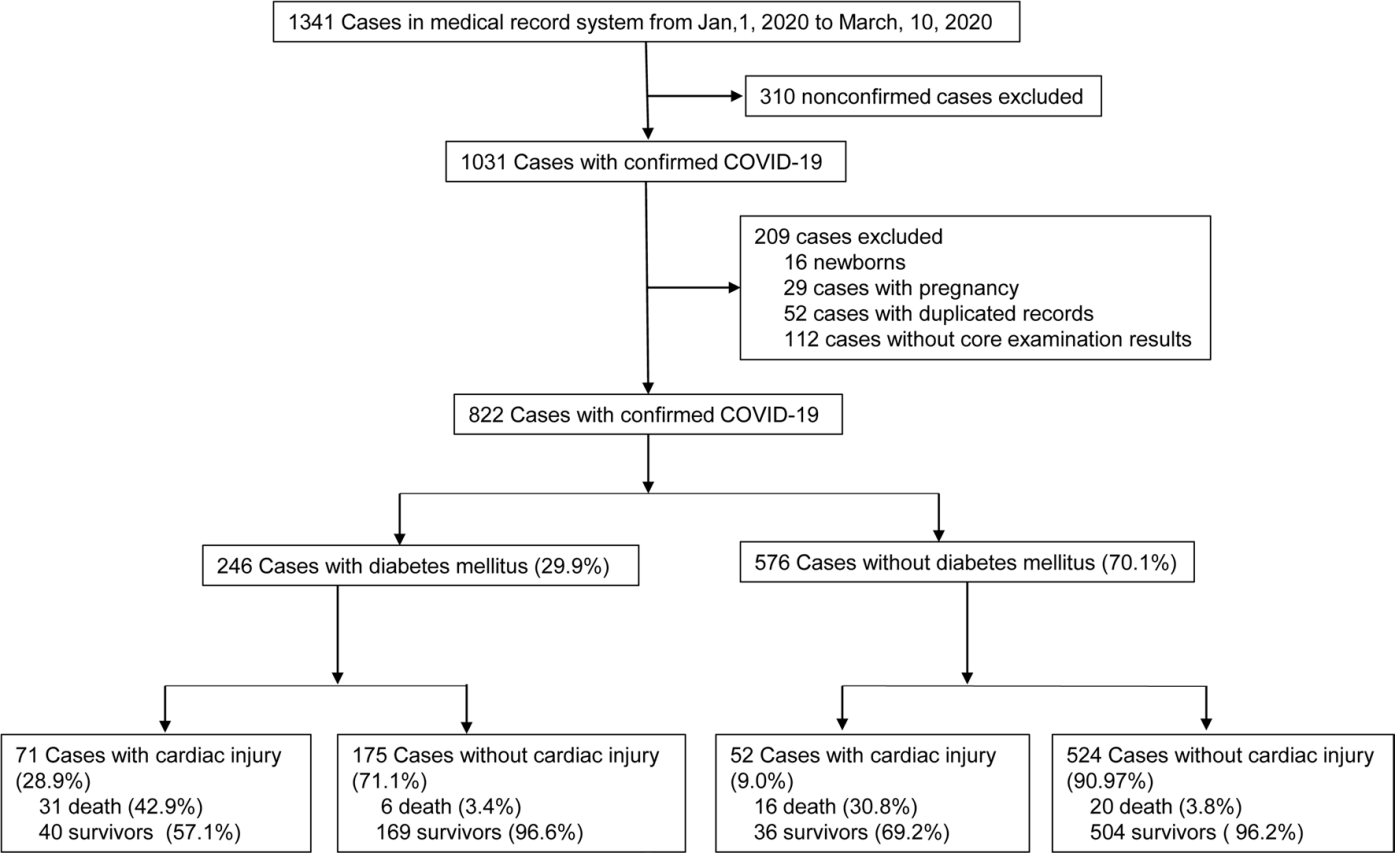
Flowchart of the study. COVID-19 = The Coronavirus disease 2019; *DM* diabetes mellitus

A total of 1341 cases were screened initially from 1 January to 10 March 2020 in the study (Fig. 1). After excluding 310 suspected patients with COVID-19, 52 duplicated cases, 16 neonates, 29 women with pregnancy, and 112 persons without available core medical information, there are 822 cases with confirmed COVID-19 were enrolled in the final analysis. Of these, 246 patients (29.93%) were diagnosed with DM. The characteristics of 112 patients without core medical information were shown in Additional file 1: Table S1.

### Baseline features of COVID-19 patients with and without DM

As shown in Table 1, when compared to patients without DM, diabetic cases were older (median age was 67 years vs 60.5 years, *p* < 0.001), more underlying coexisting comorbidities, especially hypertension (34.2% vs 13.9%, *p* < 0.001) and coronary heart disease (CHD) (11.8% vs 7.5%, *p* = 0.045), and the less manifestation of ground-glass opacity (67.7% vs 76.5%, *p* = 0.016). The initial symptoms were similar between the two groups except for sore throat or throat discomfort (6.1% in no-DM patients and 2.0% in DM, *p* = 0.014). The mean days from illness onset to hospitalization were both 10 days in DM and non-DM patients (*p* = 0.505).

**Table 1:** The demographic features, treatments, laboratory findings, and clinical outcomes of COVID-19 patients with and without DM

Characteristics	Without DM				With DM				*p* value
	All (A)	non–cardiac injury	cardiac injury	*p* value	All (B)	non–cardiac injury	cardiac injury	*p* value	A vs. B
n (%)	576 (100.0)	524 (91.0)	52 (9.0)	–	246 (100.0)	175 (71.1)	71 (28.9)	–	–
Gender, n (%)									
Female	288 (50.0)	271 (51.7)	17 (32.7)	0.009	115 (46.8)	86 (49.1)	29 (40.9)	0.237	0.393
Male	288 (50.0)	253 (48.3)	35 (67.3)	–	131 (53.3)	89 (50.9)	42 (59.2)	–	–
Age (IQR)	60.5 (49.0–69.0)	59.0 (47.3–67.0)	76.5 (68.0–83.0)	< 0.001	67.0 (57.0–74.0)	64.0 (57.0–71.0)	74.0 (65.0–81.0)	< 0.001	< 0.001
Time from onset to hospital admission (IRQ), days	10.0 (7.0–14.0)	10.0 (7.0–14.0)	10.0 (6.3–14.0)	0.117	10. (7.0–15.0)	10.0 (7.0–15.0)	10.0 (7.0–15.0)	0.797	0.505
SPO2 (%)	97.0 (93.0–99.0)	97.0 (94.0–99.0)	93.5 (81.8–98.0)	0.008	96.0 (91.0–98.0)	97.0 (93.0–99.0)	93.0 (85.0–97.0)	< 0.001	0.022
RR (beats per minute)	20.0 (18.0–21.0)	20.0 (18.0–21.0)	20.0 (19.0–24.6)	0.068	20.0 (19.0–23.0)	20.1 (19.0–21.0)	21.0 (18.0–26.0)	0.485	0.027
Pulse rate (beats per minute)	85 (78–97)	85 (78–97)	88 (78–102)	0.875	87 (79–101)	86 (79–100)	89 (78–102.5)	0.054	0.326
SBP (mmHg)	125 (115–136)	125 (115–135)	130.5 (112.5–150)	0.137	130 (118.25–143)	129 (119–141)	129 (116–150)	0.858	0.001
DBP (mmHg)	76 (68–82.5)	76 (68–82)	75 (66–87.8)	0.293	77 (70–84)	77 (70–84)	76 (68–87)	0.839	0.047
Symptoms, n (%)									
Asymptomatic	14 (2.4)	11 (2.0)	3 (5.8)	0.06	10 (4.1)	6 (3.4)	4 (5.6)	0.48	0.202
Fever	460 (79.9)	425 (81.1)	35 (67.3)	0.018	207 (84.2)	149 (85.1)	58 (81.7)	0.502	0.15
Dry cough	361 (62.7)	331 (63.2)	30 (57.7)	0.436	139 (56.5)	103 (58.9)	36 (50.7)	0.178	0.097
Sputum production	137 (23.8)	122 (23.3)	15 (28.9)	0.369	45 (18.3)	29 (16.6)	16 (22.5)	0.273	0.082
Fatigue	196 (34.4)	179 (34.2)	17 (32.7)	0.832	77 (31.3)	57 (32.6)	20 (28.2)	0.5	0.447
Myalgia	50 (8.7)	47 (9.0)	3 (5.8)	0.434	16 (6.5)	12 (6.9)	4 (5.6)	1	0.293
Dyspnoea/pant	212 (36.8)	191 (36.5)	21 (40.4)	0.575	80 (32.5)	60 (34.3)	20 (28.2)	0.353	0.24
Vomiting/diarrhea/nausea	25 (4.3)	23 (4.4)	2 (3.9)	1	18 (7.3)	16 (9.1)	2 (2.8)	0.084	0.079
Abdominal pains/diarrhea	80 (13.9)	73 (13.9)	7 (13.5)	0.926	36 (10.4)	29 (16.6)	7 (9.9)	0.177	0.779
Sore throat/throat discomfort	35 (6.1)	31 (5.9)	4 (7.7)	0.609	5 (2.0)	5 (2.9)	0 (0.0)	0.325	0.014
Headache/dizziness	33 (5.7)	32 (6.1)	1 (1.9)	0.347	14 (5.7)	11 (6.3)	3 (4.2)	0.763	0.983
Chest distress	138 (24.0)	131 (25.0)	7 (13.5)	0.063	58 (23.6)	44 (25.1)	14 (19.7)	0.364	0.907
Coexisting comorbidities, n (%)									
Any	226 (39.2)	187 (35.7)	39 (75.0)	< 0.001	160 (65.0)	109 (62.3)	51 (71.8)	0.155	< 0.001
Hypertension	80 (13.9)	61 (11.6)	19 (36.5)	< 0.001	84 (34.2)	61 (34.9)	23 (32.4)	0.712	< 0.001
Coronary heart diseases	43 (7.5)	35 (6.7)	8 (15.4)	0.045	29 (11.8)	17 (9.7)	11 (15.5)	0.196	0.045
Cancer	17 (3.0)	14 (2.7)	3 (5.8)	0.192	8 (3.3)	6 (3.4)	2 (2.8)	1	0.818
Pulmonary diseases	31 (5.4)	24 (4.6)	7 (13.5)	0.016	17 (7.0)	8 (4.6)	9 (12.7)	0.048	0.392
Cerebrovascular diseases	14 (2.4)	8 (1.5)	6 (11.5)	0.001	12 (4.9)	4 (2.3)	8 (11.3)	0.006	0.066
Laboratory findings									
FPG (mmol/l)	5.2 (4.7–6.0)	5.2 (4.7–6.0)	5.4 (4.5–6.4)	0.956	7.5 (5.8–10.1)	7.4 (5.7–10.0)	7.8 (5.8–10.2)	0.748	< 0.001
ALT (U/l)	38.0 (21.0–65.0)	38.0 (21.0–66.0)	35.0 (21.0–56.0)	0.378	39.0 (21.8–73.3)	40.0 (22.0–74.0)	38.0 (21.0–73.0)	0.55	0.637
AST (U/l)	34.0 (24.0–51.0)	34.0 (23.0–49.0)	48.0 (34.0–57.0)	0.172	36.0 (23.0–61.0)	32.0 (22.0–48.0)	52.0 (32.0–79.0)	0.092	0.205
Albumin (g/l)	34.8 (31.6–37.6)	35.1 (32.3–37.7)	31.0 (27.9–32.7)	< 0.001	32.1 (28.8–34.9)	33.4 (30.4–35.8)	28.4 (25.0–31.9)	< 0.001	< 0.001
Urea (mmol/l)	5.3 (4.3–6.8)	5.2 (4.2–6.6)	7.6 (6.5–9.7)	0.021	7.0 (5.4–9.2)	6.2 (5.2–7.8)	9.4 (7.6–18.8)	< 0.001	< 0.001
Creatinine (µmol/l)	64.0 (53.0–76.0)	63.0 (53.0–75.0)	73.0 (61.0–99.0)	0.179	67.5 (56.8–85.3)	65.0 (55.0–78.0)	78.0 (65.0–98.0)	0.07	0.002
Platelet (× 10^9^/l)	214.0 (164.0–272.3)	217.0 (166.0–276.0)	180.5 (145.5–229.8)	0.001	209.0 (150.5–275.5)	220.0 (165.0–289.0)	177.0 (119.0–20.0)	< 0.001	0.174
APTT (sec)	25.9 (24.2–28.1)	25.9 (24.2–27.9)	26.8 (24.7–28.1)	0.374	25.7 (24.0–27.4)	25.5 (24.0–27.1)	26.4 (24.1–28.7)	0.466	0.129
PT (sec)	12.0 (11.5–12.7)	12.0 (11.4–12.7)	12.7 (12.0–14.2)	< 0.001	12.4 (11.8–13.4)	12.1 (11.6–12.8)	13.4 (12.6–16.4)	0.015	< 0.001
TT (sec)	17.1 (16.4–17.8)	17.1 (16.4–17.8)	16.9 (16.0–18.0)	0.92	16.8 (15.9–17.7)	16.9 (16.1–17.7)	16.4 (15.5–17.6)	0.828	0.002
INR	1.0 (1.0–1.1)	1.0 (1.0–1.1)	1.1 (1.0–1.2)	< 0.001	1.1 (1.0–1.2)	1.1 (1.0–1.1)	1.2 (1.1–1.4)	0.019	< 0.001
D–dimer (mg/l)	1.1 (0.5–3.4)	1.0 (0.4–2.7)	4.5 (1.5–8.5)	0.026	2.4 (0.9–6.7)	1.4 (0.7–4.2)	8.1 (4.0–9.8)	< 0.001	< 0.001
Fibrinogen (g/l)	4.6 (3.6–5.7)	4.6 (3.5–5.7)	4.6 (3.8–5.6)	0.997	16.8 (15.9–17.7)	5.0 (4.2–6.4)	5.4 (4.0–6.8)	0.987	< 0.001
TC (mmol/l)	3.8 (3.3–4.4)	3.8 (3.3–4.4)	3.8 (3.3–4.2)	0.249	3.7 (1.5–4.3)	3.8 (3.2–4.3)	3.6 (3.0–4.0)	0.127	0.189
Triglyceride (mmol/l)	1.2 (0.9–1.6)	1.2 (0.9–1.6)	1.3 (0.9–1.5)	0.926	1.3 (1.0–1.7)	1.3 (1.0–1.7)	1.3 (1.1–1.7)	0.992	0.001
HDL–c (mmol/l)	0.9 (0.8–1.1)	0.9 (0.8–1.1)	0.9 (0.7–1.1)	0.559	0.9 (0.7–1.0)	0.9 (0.7–1.0)	0.8 (0.7–1.0)	0.334	< 0.001
LDL–c (mmol/l)	2.3 (1.9–2.9)	2.4 (1.9–2.9)	2.2 (1.9–2.6)	0.097	2.3 (1.7–2.8)	2.4 (1.8–2.9)	2.1 (1.7–2.6)	0.032	0.18
cTnI (ng/ml)	0.003 (0.003–0.012)	0.003 (0.003–0.007)	0.104 (0.059–0.297)	< 0.001	0.011 (0.003–0.057)	0.003 (0.003–0.012)	0.159 (0.070–0.757)	< 0.001	< 0.001
CK–MB (ng/ml)	0.9 (0.6–1.4)	0.8 (0.6–1.3)	2.1 (1.3–3.3)	< 0.001	1.3 (0.8–2.4)	0.99 (0.73–14.14)	2.96 (1.69–4.26)	< 0.001	< 0.001
Myoglobin (µg/l)	36.9 (25.5–59.9)	35.3 (25.0–54.3)	111.3 (46.4–255.5)	< 0.001	54.3 (33.8–98.6)	45.0 (30.8–82.6)	89.9 (61.2–165.4)	< 0.001	< 0.001
BNP (pg/ml)	89.9 (42.4–300.9)	79.5 (38.0–196.1)	626.0 (324.0–1467.0)	< 0.001	250.0 (79.0–779.0)	131.7 (68.0–452.4)	877.0 (454.0–5528.0)	< 0.001	< 0.001
Findings on chest CT, n/N (%)									
Unilateral	17/467 (3.6)	14/432 (3.2)	3/35 (8.6)	0.111	2/190 (1.1)	1/146 (0.7)	1/44 (2.3)	0.41	0.069
Bilateral	444/467 (95.1)	412/432 (95.4)	32/35 (91.4)	–	188/190 (99.0)	145/146 (99.3)	43/44 (97.7)	–	–
Ground–glass opacity	357/467 (76. 5)	333/432 (77.1)	24/35 (68.6)	0.254	128/190 (67.4)	109/146 (74.7)	19/44 (43.2)	< 0.001	0.016
Treatment, n (%) or n/N (%)									
Antiviral therapy	521 (90. 5)	477 (91.0)	44 (84.6)	0.138	229 (93.1)	164 (93.7)	65 (91.6)	0.582	0.221
Antibiotic therapy	418 (72.6)	376 (71.8)	42 (80.8)	0.165	203 (82.5)	135 (77.1)	68 (95.8)	< 0.001	0.002
Invasive mechanical ventilation	31 (5.4)	24 (4.6)	7 (13.5)	0.016	54 (22.0)	24 (13.7)	30 (42.3)	< 0.001	< 0.001
Glucocorticoid therapy	249 (43.2)	221 (42.2)	28 (53.9)	0.105	145 (58.9)	93 (53.1)	52 (73.2)	0.004	< 0.001
Only insulin therapy	–	–	–	–	73/168 (43.5)	39/120 (32.5)	34/48 (70.8)	< 0.001	–
Insulin and OAH therapy	–	–	–	–	63/168 (37.5)	51/120 (42.5)	12/48 (25.0)	< 0.001	–
Only OAH therapy	–	–	–	–	32/168 (19.1)	30/120 (25.0)	2/48 (14.3)	< 0.001	–
Coagulopathy, n (%)	21 (3.7)	11 (2.1)	10 (19.2)	< 0.001	47 (19.1)	22 (12.6)	25 (35.2)	< 0.001	< 0.001
Death. n (%)	36 (6.3)	20 (3.8)	16 (30.8)	< 0.001	37 (15.0)	6 (3.4)	31 (42.9)	< 0.001	< 0.001

*ALT* alanine transaminase, *APTT* activated partial thromboplastin time, *AST* aspartate transaminase, *BNP* brain natriuretic peptide, *CK-MB* creatine phosphokinase-MB, *COVID-19* the Coronavirus disease 2019, *cTnI* cardiac troponin I, *DBP* diastolic pressure, *DM* diabetes mellitus, *FPG* fasting plasma glucose, *HDL-c* high-density lipoprotein cholesterol, INR international normalized ratio, *LDL-c* low-density lipoprotein cholesterol, *OAH* oral anti-hyperglycemia, *PT* prothrombin time, *RR* respiratory rate, *SBP* systolic pressure, *TC* total cholesterol, *TG* triglycerides, *TT* thrombin time

Regarding the laboratory findings, DM patients had a relatively higher median level of creatinine, fasting plasm glucose (FPG), and D-dimer, but less SPO_2_ and albumin (all *p* < 0.05) (Table 1). For blood lipids profile, compared with non-DM individuals, patients with DM shown the higher median (IQR) value of triglyceride (1.3 (1.0–1.7) mmol/l vs 1.2 (0.9–1.6) mmol/l, *p* = 0.001) and lower high-density lipoprotein (HDL-c) (0.9 (0.7–1.0) mmol/l vs 0.9 (0.8–1.1) mmol/l, *p* < 0.001), but with similar concentration of low-density lipoprotein (LDL-c) (Table 1). The incidence of severe complications in COVID-19 patients with and without DM was also shown in Additional file 1: Fig. S1.

Among diabetic patients, people with cardiac injury were older (median age, 74 years vs 64 years, *p* < 0.001) than individuals without cardiac injury. These people also required more treatment with antibiotic agents, glucocorticoids, and invasive mechanical ventilation (all *p* < 0.05). On the hypoglycemic strategies, the use of insulin was more frequent in DM patients with cardiac injury (*p* < 0.001) (Table 1).

### Higher incidence of cardiac injury in COVID-19 patients with DM

**Fig. 2:**
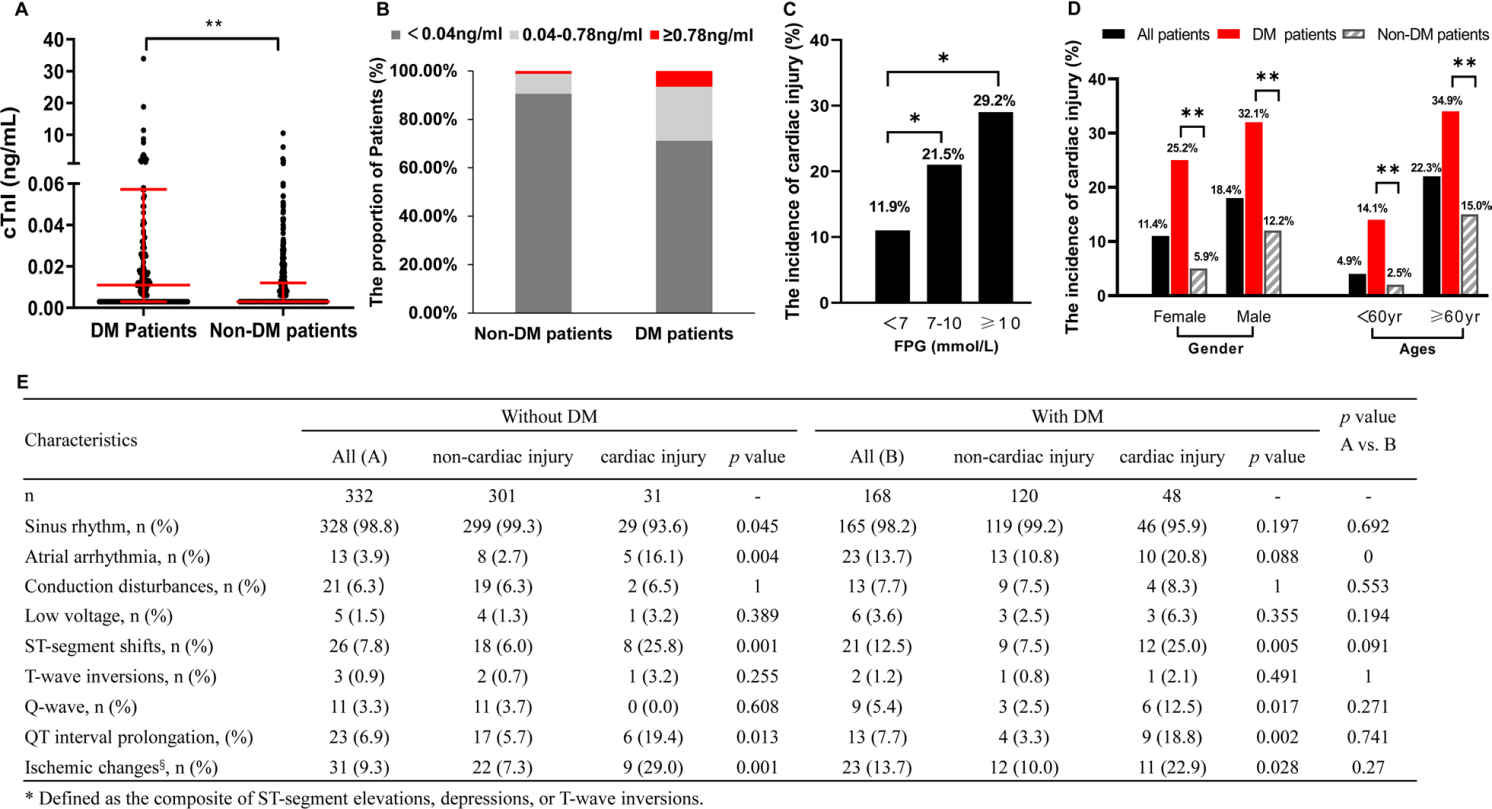
The distribution of cTnI and the electrocardiographic features of COVID-19 patients with and without diabetes. The peak levels of cTnI (**A**) are higher in patients with DM during hospitalization. The incidence of cardiac injury in COVID-19 patients is higher in DM groups according to the level of cTnI (**B**) and FPG (**C**) and age and gender (**D**). **E** Present the electrocardiographic characteristics of COVID-19 patients. *Present *p* < 0.05, **present *p* < 0.01. ^§^Defined as the composite of ST-segment elevations, depressions, or T-wave inversions. *cTnI* cardiac troponin I, *DM* diabetes mellitus, *FPG* fasting plasma glucose

Overall, the incidence of cardiac injury was higher in patients with DM than in non-DM cases (28.9% vs 9.0%, *p* < 0.001), even grouped by age, gender, and the level of FPG (Fig. 2). During hospitalization, the mean peak concentration of cTnI, creatine kinase MB (CK-MB), myoglobin (Myo), and B-type natriuretic peptide (BNP) was higher in COVID-19 patients with DM (Table 1). The distribution of cTnI showed that DM patients had a great proportion of cTnI > 0.04 ng/ml and cTnI > 0.78 ng/ml, which indicated acute myocardial infarction possible (all *p* < 0.01 with *χ*^2^ test) (Fig. 2).

Of patients with COVID-19, 500 cases (60.8%) underwent an examination of 12-lead electrocardiogram after admission. The features of electrocardiogram were similar in COVID-19 patients with or without DM, except the incidence of atrial arrhythmia (Fig. 2). However, in these two groups, patients with cardiac injury more frequently had ischemic changes, especially ST-segment shifts (elevation or depression), compared with those without myocardial infarction (all *p* < 0.05).

### Increased in-hospital mortality in DM patients with cardiac injury

**Fig. 3:**
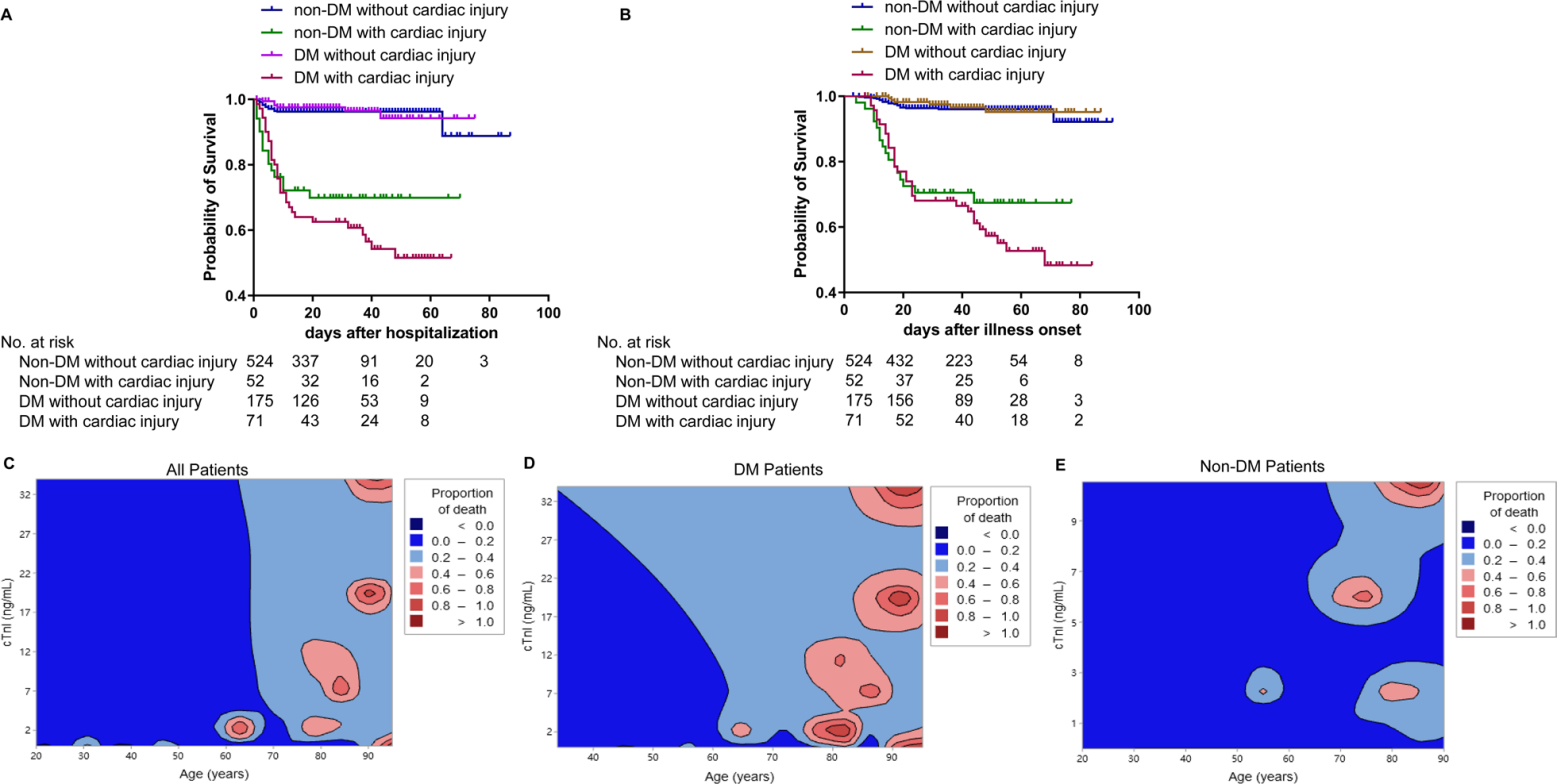
The effect of cardiac injury on mortality of COVID-19 with and without DM. Kaplan–Meier survival curves showed higher mortality in patients with DM and cardiac injury by the days after hospitalization (**A**) and illness onset (**B**). The contour plot revealed the mortality was higher in patients of senior age and elevated cTnI level in all COVID-19 patients (**C**), DM patients (**D**), and non-DM patients (**E**). *cTnI* cardiac troponin I, *DM* diabetes mellitus

The overall in-hospital mortality in patients with and without DM was 37/246 (15.0%) and 36/576 (6.3%), respectively (*p* < 0.001). It was also higher in people with cardiac injury, both in DM patients (42.9% vs 3.4%, *p* < 0.001) and in non-DM patients (32.1% vs 3.8%, *p* < 0.001) (Figs. 1 and 3A, B). The contour plot shows the elevated fatality rate was closely related to the elderly and higher levels of cTnI (Fig. 3C–E).

### Severer immune-inflammatory responses in DM patients with cardiac injury

As compared to non-DM cases, COVID-19 with DM patients showed relatively higher median counts of white blood cells (WBC) and Neutrophils (NEU), but a lower number of lymphocytes (LYM), which led to a higher level of neutrophil to lymphocyte ratio (NLR) (5.9 (3.0–10.5) vs 3.0 (1.9–5.4), *p* < 0.001). These subjects also present higher levels of IL-6 (10.7 (5.5–25.1) vs 6.0 (3.1–11.7) pg/ml, *p* < 0.001) and IL-10 (6.3 (5.1–8.0) vs 5.5 (4.7–6.7) pg/ml, *p* < 0.001). Moreover, the absolute counts of all immunocytes were decreased in DM patients compared to non-DM cases, including CD3^+^ T cells, CD3^+^CD4^+^ T cells counts, CD3^+^CD8^+^ T cells, CD19^+^ B cells, and CD16^+^CD56^+^ NK cells (all *p* < 0.05). The concentrations of immunoglobulin A (IgA) and IgG were also risen in DM patients (all *p* < 0.01) (Table 2).

**Table 2:** The immunological features of COVID-19 patients with and without cardiac injury grouped by DM

Characteristics	Without DM				With DM				*p* value
	All (A)	non-cardiac injury	cardiac injury	*p* value	All (B)	non-cardiac injury	cardiac injury	*p* value	A vs. B
C3 (g/l)	1.0 (0.9–1.2)	1.0 (0.9–1.2)	1.0 (0.9–1.1)	0.287	1.0 (0.9–1.1)	1.0 (0.9–1.2)	0.9 (0.8–1.1)	0.001	0.208
C4 (g/l)	0.3 (0.2–0.3)	0.3 (0.2–0.3)	0.3 (0.2–0.3)	0.015	0.3 (0.2–0.3)	0.3 (0.2–0.3)	0.3 (0.2–0.3)	0.209	0.23
IgA (g/l)	2.3 (1.7–2.9)	2.3 (1.7–2.9)	2.4 (2.0–3.0)	0.082	2.7 (2.0–3.6)	2.6 (1.9–3.4)	3.1 (2.1–3.8)	0.065	< 0.001
IgE (IU/ml)	42.0 (9.2–106.3)	42.0 (9.2–107.8)	40.5 (9.2–90.5)	0.928	54.0 (9.2–13.0)	54.0 (9.2–143.0)	50.5 (9.2–95.8)	0.289	0.157
IgG (g/l)	11.7 (10.0–13.9)	11.7 (10.0–13.9)	11.8 (9.6–13.9)	0.835	12.3 (10.4–14.9)	12.1 (10.1–14.7)	12.8 (11.4–15.1)	0.205	0.009
IgM (g/l)	0.9 (0.7–1.2)	0.9 (0.7–1.2)	0.9 (0.6–1.2)	0.249	0.9 (0.7–1.3)	0.9 (0.7–1.2)	1.0 (0.8–1.4)	0.069	0.695
WBC (× 10^9^/l)	5.2 (3.9–7.0)	5.1 (3.9–6.8)	6.2 (4.4–10.0)	0.001	6.2 (4.7–8.3)	5.8 (4.5–7.6)	7.0 (5.2–11.4)	0.008	< 0.001
LYM (× 10^9^/l)	1.1 (0.8–1.5)	1.1 (0.8–1.5)	0.8 (0.6–1.3)	< 0.001	0.9 (0.6–1.2)	1.0 (0.6–1.3)	0.7 (0.5–1.0)	< 0.001	< 0.001
NEU (× 10^9^/l)	3.3 (2.4–5.1)	3.3 (2.3–4.8)	4.7 (3.0–7.8)	< 0.001	4.7 (3.3–6.9)	4.2 (2.8–6.1)	5.7 (4.2–9.5)	< 0.001	< 0.001
Baso (× 10^9^/l)	0.01 (0.01–0.02)	0.01 (0.01–0.02)	0.01 (0.01–0.02)	0.332	0.01 (0.01–0.03)	0.01 (0.01–0.03)	0.01 (0.01–0.02)	0.137	0.196
EOS (× 10^9^/l)	0.02 (0.00–0.06)	0.02 (0.00–0.06)	0.01 (0.00–0.04)	0.226	0.01 (0.00–0.06)	0.01 (0.00–0.08)	0.00 (0.00–0.01)	< 0.001	0.032
CD3 + T cells (cells/ul)	657.5 (442.5–940.5)	677.0 (457.5–962.5)	452.0 (249.0–619.0)	< 0.001	480.0 (283.5–79.0)	558.0 (322.0–844.0)	338.0 (237.8–41.4)	< 0.001	< 0.001
CD3 + CD4 + T cells (cells/ul)	392.5 (246.5–576.0)	407.0 (260.5–596.5)	265.0 (158.0–414.0)	< 0.001	292.0 (182.5–84.0)	365.0 (193.5–35.0)	203.0 (125.3–16.0)	< 0.001	0.033
CD3 + CD8 + T cells (cells/ul)	223.0 (138.0–334.8)	228.0 (147.0–338.0)	165.0 (74.0–232.0)	< 0.001	149.0 (80.0–260.5)	176.0 (100.5–263.5)	110.0 (59.0–204.3)	< 0.001	< 0.001
CD4/CD8 ratio	1.8 (1.2–2.6)	1.8 (1.3–2.5)	1.8 (1.1–2.7)	0.942	1.9 (1.3–2.9)	1.9 (1.3–2.8)	2.0 (1.2–3.5)	0.693	0.033
CD19 + B cells (cells/ul)	135.0 (90.0–205.0)	138.0 (92.0–206.0)	112.0 (66.0–158.0)	0.004	119.0 (77.5–189.5)	131.0 (83.5–225.0)	102.5 (60.0–169.8)	0.007	0.044
CD16 + CD56 + NK cells (cells/ul)	118.0 (77.0–185.0)	117.0 (77.0–183.0)	145.0 (59.0–252.0)	0.438	109.0 (63.0–171.0)	115.0 (68.0–176.5)	79.0 (45.5–170.8)	0.057	0.018
IL2 (pg/ml)	3.8 (3.4–4.1)	4.0 (3.0–4.0)	4.0 (3.0–4.0)	0.542	3.7 (3.3–4.1)	4.0 (3.0–4.0)	4.0 (3.0–4.0)	0.659	0.516
IL4 (pg/ml)	3.4 (3.0–3.9)	4.0 (3.0–4.0)	4.0 (3.0–4.0)	0.663	3.4 (3.0–3.8)	4.0 (3.0–4.0)	4.0 (3.0–4.0)	0.337	0.604
IL5 (pg/ml)	2.2 (2.1–2.3)	2.2 (2.1–2.3)	2.3 (2.1–2.3)	0.992	2.2 (2.1–2.4)	2.3 (2.2–2.4)	2.0 (2.0–2.3)	0.19	0.804
IL6 (pg/ml)	6.0 (3.1–11.7)	6.0 (3.0–11.0)	9.5 (5.0–22.3)	0.007	10.7 (5.5–25.1)	8.0 (5.0–19.0)	25.0 (13.8–90.0)	< 0.001	< 0.001
IL10 (pg/ml)	5.5 (4.7–6.7)	6.0 (5.0–7.0)	6.0 (5.0–8.5)	0.022	6.3 (5.1–8.0)	6.0 (5.0–8.0)	8.0 (6.0–9.0)	< 0.001	< 0.001
TNF-α (pg/ml)	3.4 (2.9–4.7)	3.0 (3.0–5.0)	3.0 (3.0–4.0)	0.35	3.3 (2.9–5.7)	3.0 (3.0–6.0)	3.0 (3.0–5.3)	0.611	0.447
γ-interferon (pg/ml)	3.5 (3.0–4.3)	3.0 (3.0–4.0)	3.0 (3.0–4.0)	0.001	3.4 (3.0–4.3)	3.0 (3.0–4.0)	3.5 (3.0–4.3)	0.008	< 0.001
CRP (mg/l)	4.8 (2.2–28.4)	4.0 (2.0–21.0)	36.0 (7.0–65.0)	0.009	4.4 (2.3–51.9)	4.0 (2.0–32.4)	39.0 (4.0–196.0)	0.032	0.567
PCT (ng/ml)	0.1 (0.0–0.10)	0.0 (0.0–0.0)	0.0 (0.0–0.0)	< 0.001	0.1 (0.1–0.3)	0.0 (0.0–0.0)	0.0 (0.0–1.0)	< 0.001	< 0.001
NLR	3.0 (1.9–5.4)	2.8 (1.8–5.1)	5.1 (3.5–12.5)	< 0.001	5.9 (3.0–10.5)	4.3 (2.6–9.3)	8.8 (6.3–14.7)	< 0.001	< 0.001

*Baso* basophils, *C3* complement 3, *C4* complement 4, *CRP* C-reaction protein, *DM* diabetes mellitus, *EOS* eosinophils, *IgA* immunoglobulin A, *IgE* immunoglobulin E, *IgG* immunoglobulin G, *IgM* immunoglobulin M, *IL-2* interleukin-2, *IL-4* interleukin-4, *IL-5* interleukin-5, *IL-6* interleukin-6, *IL-10* interleukin-10, *LYM* lymphocytes, *NLR* neutrophils to lymphocytes ratio, *PCT* procalcitonin, *TNF-α* tumor necrosis factor-α, *WBC* white blood cells

COVID-19 patients with DM and cardiac injury had a further decreased count of LYM and increased number of Neutrophils and relatively higher NLR than those without cardiac injury. The inflammation-related biomarkers were also augmented in COVID-19 patients with cardiac injury more than those without cardiac injury. In addition, the decreased level of C3 was a specific character present in DM with cardiac injury, while the increased value of C4 was more distinctive in non-DM patients with cardiac injury (Table 2). Regarding the immunocyte subset, we also found the descending absolute counts of immune cells in cardiac injury groups (all *p* < 0.01) (Table 2). However, when compared with cases with cardiac injury but without DM, diabetic people with cardiac injury had a relatively higher frequency of CD3^+^ T cells, CD3^+^CD4^+^ T cells, and CD19^+^ B cells (Additional file 1: Fig. S2).

### Correlation between immune-inflammatory indicators and cTnI in DM patients

**Fig. 4:**
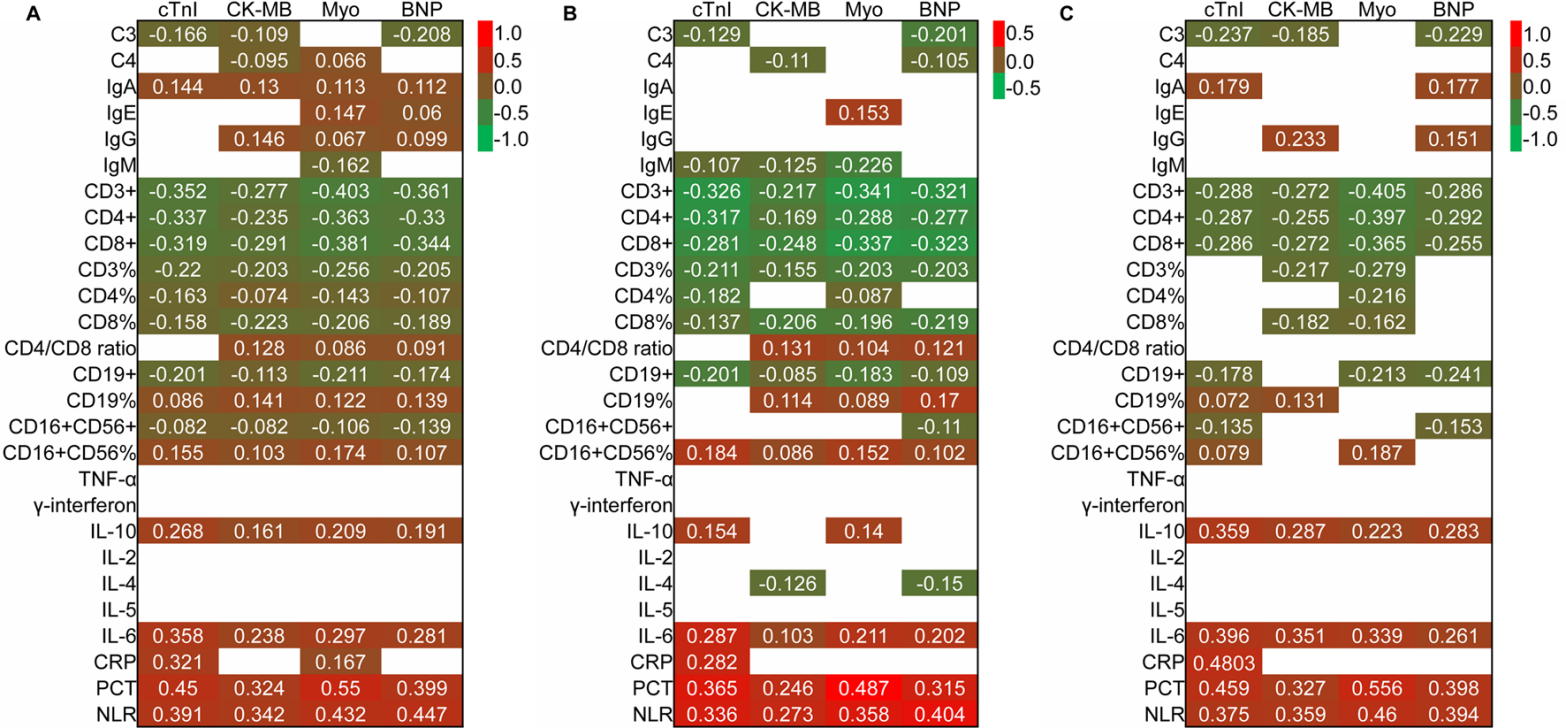
The correlation between immune-inflammatory parameters and cardiac injury. Spearman’s correlation of the contribution of immune-inflammatory response to cardiac injury is analyzed in all COVID-19 patients (**A**), non-DM patients (**B**), and DM patients (**C**). The color and number key represent the significant regression coefficients of the variables, while the blank means no statistical difference. *BNP* B type natriuretic peptide, *C3* complement 3, *C4* complement 4, *CK-MB* creatine kinase MB, *CRP C* reaction protein, *cTnI* cardiac troponin I, *DM* diabetes mellitus, *IgA* immunoglobulin A, *IgE* immunoglobulin E, *IgG* immunoglobulin G, *IgM* immunoglobulin M, *IL-2* interleukin-2, *IL-4* interleukin-4, *IL-5* interleukin-5, *IL-6* interleukin-6, *IL-10* interleukin-10, *Myo* myoglobin, *NLR* neutrophils to lymphocytes ratio, *PCT* procalcitonin, *TNF-α* tumor necrosis factor-α

In DM patients, spearman’s correlation analysis found negative connection between cardiac injury with C3 concentration (*r* = − 0.237), CD3^+^ T cells counts (*r* = − 0.288), CD3^+^CD4^+^ T cells counts (*r* = − 0.287), CD3^+^CD8^+^ T cells counts (*r* = − 0.286), CD19^+^ B cells counts (*r* = − 0.178), CD16^+^CD56^+^ NK cells counts (*r* = − 0.135), and positive association with IgA concentration (*r* = 0.179), the proportion of CD19^+^ B cells (*r* = 0.072), CD16^+^CD56^+^ NK cells (*r* = 0.079), the level of IL-10 and IL-6 (*r* = 0.359 and 0.396, respectively), and the value of CRP, PCT and NLR (*r* = 0.48, 0.459 and 0.3375, respectively) (all *p* < 0.05) (Fig. 4).

### Immune-inflammatory biomarkers as risk factors for cardiac injury in DM patients

Given the specific changes in DM groups, especially with cardiac injury, the ROC and Logistics regression analysis were performed on absolute counts of immunocyte subset, level of C3, IgA, IgG, IL-6, IL-10, γ-interferon, PCT, and NLR. The ROC curve disclosed significant cutoff levels for the immune-related biomarkers that were statistically related with in-hospital mortality in all participants: C3 ≤ 1.05 g/l; IgA > 3.18 g/l; IgG > 10.5 g/l; CD3^+^ T cells counts ≤ 333 cells/μl; CD3^+^CD4^+^ T cells counts ≤ 288 cells/μl; CD3^+^CD8^+^ T cells counts ≤ 188 cells/μl; CD19^+^ B cells counts ≤ 102 cells/μl; CD16^+^CD56^+^ NK cells ≤ 59 cells/μl; IL-6 > 25.68mpg/ml; IL-10 > 5.71 pg/ml; γ-interferon ≤ 3.58 ng/ml; PCT  > 0.078 ng/ml; NLR > 7.525 (Additional file 1: Fig. S3).

**Fig. 5:**
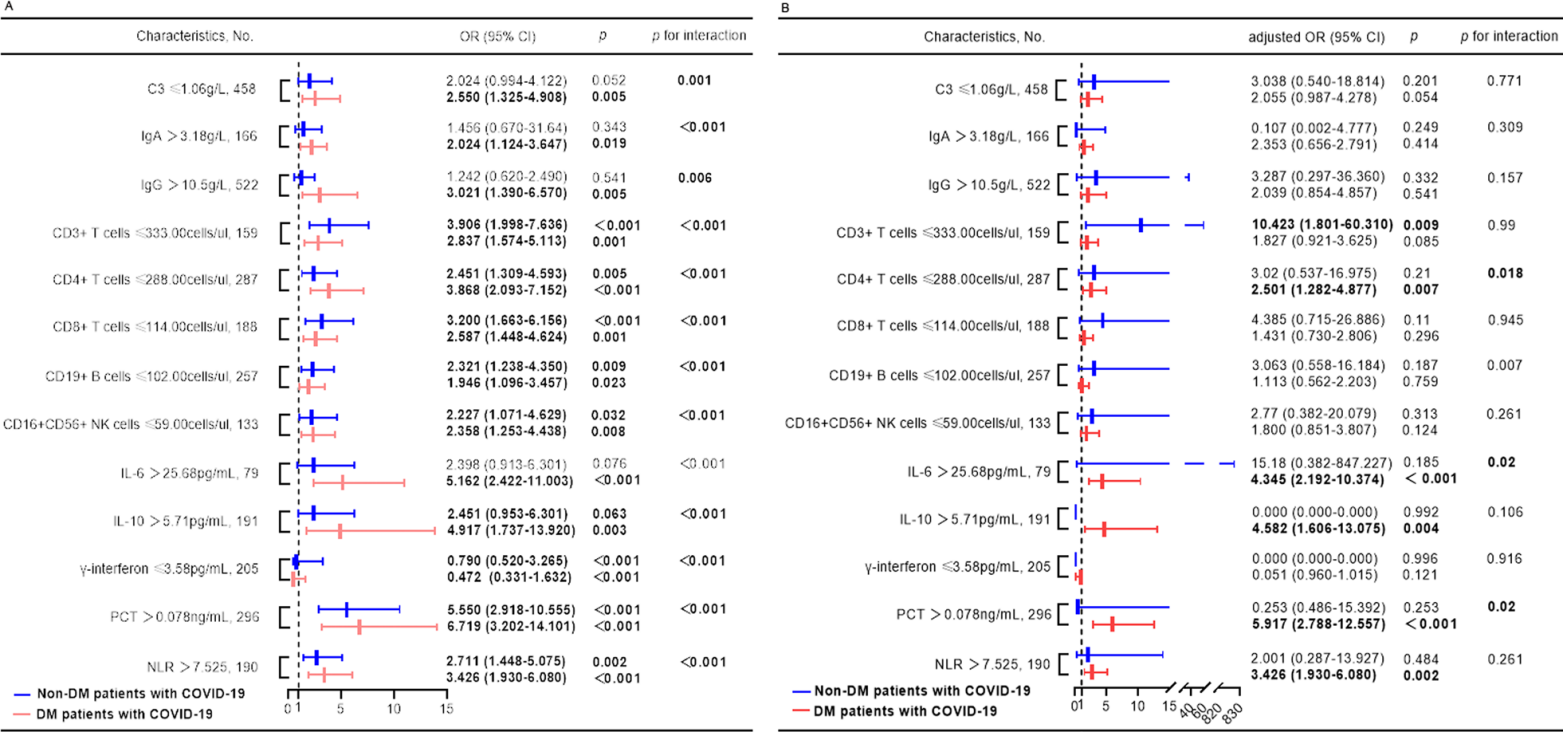
Logistics regression analysis of the risk factors of cardiac injury. The immune-inflammatory predictors of cardiac injury are present in the univariate model (**A**) and multivariate model, after adjusting age, sex, C-reaction protein, low-density lipoprotein cholesterol, and the presence of comorbidities, including hypertension, coronary heart disease, coagulopathy, and glucocorticoid therapy (**B**). Statistically significant *p* values are highlighted in bold. *C3* complement 3, *IgA* immunoglobulin A, *CI* confidence interval, *IgG* immunoglobulin G, *IL-6* interleukin-6, *IL-10* interleukin-10, *NK cells* natural killer cells, *NLR* neutrophils to lymphocytes ratio, *OR* odds ratio, *PCT* procalcitonin

To assess the risk factors for cardiac injury by logistics regression analysis, the value of these indicators was transformed into categorical variables according to the ROC cut-off point. In univariable analysis, all the above biomarkers were risk factors of cardiac injury in COVID-19 patients with DM with *P*_interaction_  < 0.01 (Fig. 5). After adjusting age, sex, CRP, LDL-c, the presence of comorbidities (included hypertension and coronary heart disease), coagulopathy, and glucocorticoid therapy, the independent predictors for cardiac injury in DM patients were IL-10 > 5.71 pg/ml (adjusted OR, 4.582; 95% CI 1.606–13.075; *p* = 0.004) and NLR > 7.525 (adjusted OR, 3.426; 95% CI 1.930–6.080; *p* = 0.002), with no detectable evidence of interaction with DM (*P*_interaction_ > 0.05). In turn, CD3^+^CD4^+^ T cells counts ≤ 288 cells/μl (adjusted OR, 2.501; 95% CI 1.282–4.877; *p* = 0.007), IL-6 > 25.68mpg/ml (adjusted OR, 4.345; 95% CI, 2.192–10.374; *p* < 0.001) and PCT  > 0.078 ng/ml (adjusted OR, 5.917; 95% CI, 2.788–12.557; *p* < 0.001) were significantly associated with cardiac injury in diabetic patients with *P*_interaction_  < 0.05 (Fig. 5).

## Discussion

In this retrospective study of 822 COVID-19 cases, three major observations were demonstrated: (i) cardiac injury was prevalent in diabetic patients with COVID-19, and conferred a nearly 13-fold higher risk of in-hospital mortality in those people; (ii) COVID-19 patients with DM and cardiac injury present much severer immune-inflammatory responses; and (iii) decreased number of CD3^+^CD4^+^ T cells and increased IL-6 value particularly refer high risk of cardiac injury in diabetic COVID-19 patients.

Consistent with previous studies [3–6], the higher overall in-hospital mortality was found in COVID-19 patients with DM (15.0% vs 6.3%, *p* < 0.001) (Fig. 1). However, studies from France and England did not support this hypothesis [21, 22]. Notably, the mean age of participants in these two studies(> 71.2 years) was older than our study (< 67 years). The older age groups always mean a higher incidence of mortality in the present study (Figs. 2 and 3) and other studies [3, 5, 6, 13]. This might prominently overestimate the mortality of COVID-19, and cover the potential effects caused by DM.

Previous reports have demonstrated that cardiac injury caused by COVID-19 can lead to poor clinical outcomes [5, 9–11]. In the present study, the risk of in-hospital death for diabetic COVID-19 patients with higher cTnI was nearly 13-fold higher than without cardiac injury (42.9% vs 3.4%, p < 0.001). When excluding patients with cardiac injury, the incidence of death was similar between DM and non-DM participants (3.4% vs 3.8%, *p* = 0.113). This means that cardiac injury also increases the risk of in-hospital mortality from DM in COVID-19 patients.

Despite cardiac injury is prevalent in diabetic patients with COVID-19 (Fig. 1) [7, 8], the etiology and risk factors of cardiac injury are not clear yet. It has been reported that basal cTnI levels were slightly increased in DM with coronary heart disease [23]. However, cTnI values in those patients were not up to the 99th percentile URL [23]. Among diabetic patients with a normal level of cTnI, the probability of being free of future cardiovascular diseases at follow-up was 92.2%. Once they have elevated cTnI above the cutoff, the risk of cardiovascular diseases was significantly increased [24]. In addition, the basal cTnI levels were lower in women than men in the general population [25], however, it was similar between diabetic men and diabetic women [26]. Kimenai et al. found the doubling of cardiovascular risk required similar thresholds of cTnI value in women and men (2.1 ng/l vs. 2.5 ng/l). The discrimination of cTnI for the prediction of cardiovascular events between women and men was also attenuated [25]. Therefore, the criterion of cardiac injury in this study was based on a single cTnI 99th percentile cutoff but not sex-specific cutoffs. Our results showed that the incidence of cardiac injury was significantly augmented both in male and female diabetic COVID-19 patients (Fig. 2).

Acute viral infections are associated with a higher risk of acute cardiac injury, ischemia, and infarction [27]. In keeping with this, cardiac pathological examinations have found cardiomyocyte hypertrophy, the infiltration of immunocytes, and possible myocardial localization of viral particles [16, 17, 20]. Among COVID-19 patients with cardiac injury, nearly 14.7%-25.8% had ST segment shift in 12-lead electrocardiogram (the common features of acute coronary syndrome) (Fig. 2E), while 63.2%-78.3% present echocardiographic abnormalities [9, 28]. Meanwhile, 38.7% of COVID-19 patients with poor outcomes had possible myocarditis [29]. These indicated that the above diseases accounted for the major causes of cardiac injury following COVID-19 infection. But the next important question is why.

One of the answers is the immunity function disturbance after COVID-19 infection. COVID-19 Patients with cardiac injury have increased inflammatory markers, such as IL-6, lymphopenia, and higher counts of leukocytes and neutrophils [10, 11, 13, 15, 20]. Trends of these changes were far graver in diabetic COVID-19 patients with cardiac injury (Table 2 and Additional file 1: Fig. S2.). What’s more, lower C3 level and increased IgA were positively associated with cTnI value in these people (Fig. 4). SARS-CoV-2 viral entry facilitated by DM can induce early mucosal immunity to generate serum IgA antibodies [30]. The consequence of increased IgA is leading to IL-6 mediated inflammatory effects [31]. Meanwhile, the SARS-CoV-2 virus may directly clip C3 to make C3a, namely complement activation, and then accelerates the development of neutrophils-induced thrombosis, coagulopathy, and tissue injury [32]. Thus, complement system and IgA-mediated mucosal immunity may involve the initiation of cardiac injury in COVID-19 patients with DM.

In particular, this study demonstrated that the decreased CD3^+^CD4^+^ T cells (≤ 288cells/ul) were an independent risk of cardiac injury in DM patients with COVID-19 (adjusted OR, 2.501; 95% CI, 1.282–4.877; *p* = 0.007; *P*_interaction_ = 0.018), but not in patients without DM (Fig. 5). It has been reported that lower blood lymphocyte percentage was an independent risk factor of cardiac injury in COVID-19 patients [15]. However, this study based on small sample sizes was not specifically designed for diabetic COVID-19 patients [15]. Actually, the impaired immune state in diabetes is characterized by an initial interruption in the activation of Th1 CD4+ T cell-mediated immunity and late hyperimmune response, which may contribute to cytokine storm dominated by IL-6 [33]. In diabetic mice infected by Middle East respiratory syndrome coronavirus (MERS-CoV), the alterations in CD4+ T cells were associated with the server and prolonged disease [34]. Consistent with this, the absolute count of T cell subsets was decreased in severe COVID-19 cases with DM [2, 14].

The present study also revealed that elevated levels of IL-6 (> 25.68 pg/ml) increase the risk of the occurrence of cardiac injury in DM persons (*P*_interaction_ < 0.05). Previous studies have investigated that higher plasma IL-6 level is an independent marker for macrovascular events and mortality in type 2 diabetic patients [35, 36]. Mostly, Zhou et al. confirmed that in COVID-19 patients, CD4+ T cells, but not CD8+ T cells, NK cells, and B cells, are the main source of IL-6 production [37]. Once IL-6 is released, it not only induces apoptosis pathway and excessive exhaustion of T cells in server COVID-19 patients, but also plays a pathological role in chronic inflammatory disease (including cardiovascular disease) after SARS-CoV-2 infection [38, 39]. Thus, IL-6 derived from SARS-CoV-2 specific CD4^+^ T cells is required for cardiac complications and death in COVID-19 patients with DM.

This study found higher IL-10, an anti-inflammatory cytokine, was associated with cardiac injury (Table 1 and Fig. 5). This is similar to previous studies [40–42]. Actually, IL-10 can predict disease severity and poor outcomes in CVOID-19 patients [40, 41]. There are some potential mechanisms to explain these results. Firstly, higher IL-10 concentrations may reflect an extreme attempt to counteract severe inflammation in COVID-19. This is because IL-10-producing regulatory T cells, which is significant increase in severe COVID-19 patients, might contribute to inhibiting innate inflammatory responses [41, 43]. Secondly, IL-10 concentrations are elevated earlier than IL-6 in COVID-19 patients [42]. IL-10 might stimulate the production of other mediators of the cytokine storm, such as IL-6, through a negative feedback mechanism [42]. Thirdly, IL-10 directly enlarges cytotoxic effector CD8+ T cells and hyperactivation of adaptive immunity to exacerbate COVID1-9 severity and tissue injury [42]. Lastly, IL-10 decreased the expression of HLA class II molecules by antigen-presenting cells [41].

Nonetheless, despite a lot of efforts we made, our study still had some notable limitations. Firstly, the single-center retrospective nature of the study leads to a lack of some data (such as the echocardiography and continuous monitoring of blood glucose and cTnI) and the absence of a prospective validation cohort. Secondly, given the continually increasing number of COVID-19 infection cases, a relatively small sample size of the study may throw doubt on the reliability of our study. Thirdly, there is no dynamic alteration of immune-inflammatory biomarkers and cardiac injury indicators in DM patients after hospitalization. At last, we no doubt have a possible selection bias in this study. Patients with chest distress and/or high risk of cardiovascular diseases, such as hypertension, were more prone to do the cTnI test for assessment of myocardial injury during the COVID-19 pandemic in our hospital. This may overestimate the rate of cardiac injury in the study.

## Conclusions

For diabetic patients with COVID-19, cardiac injury not only induced severer immune-inflammatory responses, but also increased in-hospital mortality. The decreased number of CD3^+^CD4^+^ T cells and increased level of IL-6 were independent risk factors of cardiac injury, which can be promoted by the presence of diabetes. Thus, immune-inflammatory indicators, especially CD3^+^CD4^+^ T cells and IL-6, are recommended to distinguish the people who refer to a high risk of cardiac injury and mortality from COVID-19 patients with DM. However, it remains a testable theory whether decision-making strategies based on the risk status of cardiac injury in COVID-19 patients, especially with DM, would be expected to get better outcomes.

## Supplementary Information

**Additional file 1: Table S1**. The comparison of COVID-19 patients with and without core examination results. **Figure S1.** The incidence of complications of COVID-19 patients with and without DM. **Figure S2.** The percent of immunocyte subsets in COVID-19 patients with cardiac injury grouped by DM and non-DM. **Figure S3.** ROC analysis of immune-inflammatory parameters for in-hospital mortality of all COVID-19 patients.

## Data Availability

The datasets used and/or analyzed during the current study are available from the corresponding author upon reasonable request.

## Abbreviations

C3: Complements 3
COVID-19: The Coronavirus disease 2019
cTnI: Cardiac troponin I
DM: Diabetes mellitus
IgA: Immunoglobulin A
IL-6: Interleukin-6
NK cells: Natural killer cells
NLR: Neutrophil to lymphocyte ratio
PG: Plasma glucose
SARS-CoV-2: The severe acute respiratory syndrome coronavirus 2
