# Supplementary material for: Immune-inflammatory biomarkers and the risk of cardiac injury in COVID-19 patients with diabetes: a retrospective cohort study
Source: Cardiovasc Diabetol. 2022 Sep 19;21:188. doi: 10.1186/s12933-022-01625-2 (PMC9483899; doi:10.1186/s12933-022-01625-2)
Supplement: Supplementary file 1 — Additional file 1: Table S1. The comparison of COVID-19 patients with and without core examination results. Figure S1. The incidence of complications of COVID-19 patients with and without DM. Figure S2. The percent of immunocyte subsets in COVID-19 patients with cardiac injury grouped by DM and non-DM. Figure S3. ROC analysis of immune-inflammatory parameters for in-hospital mortality of all COVID-19 patients. [file 12933_2022_1625_MOESM1_ESM.pdf]

Table S1. The comparison of COVID-19 patients with and without core examination results

Characteristics		with core examination results	without core examination results	*p* value
n (%)		822 (88.0)	112 (12.0)	-
Gender, n (%)				
	Female	403 (49.0)	61 (54.5)	0.28
	Male	419 (51.0)	51 (45.5)	-
Age (IQR)		63.0 (52.0-70.0)	52.0 (38.5-52.0)	0
Temperature (℃)		36.7 (36.4-37.0)	36.5 (36.4-36.8)	0.471
RR (beats per minute)		20.0 (18.0-22.0)	20.0 (12.0-20.0)	0.138
Pulse rate (beats per minute)		86.0 (78.0-98.0)	84.0 (78.0-99.0)	0.523
SBP (mmHg)		126.0 (116.0-138.0)	119.0 (112.0-129.0)	0.014
DBP (mmHg)		76.0 (68.0-83.0)	76.0 (65.0-80.0)	0.208
FPG (mmol/L)		5.6 (4.9-7.0)	5.2 (4.7-6.4)	0.168
SPO2 (%)		97.0 (92.0-99.0)	98.0 (96.0-98.0)	0.601
Time from onset to hospital admission (IRQ), days		10.0 (7.0-14.0)	10.0 (7.0-14.0)	0.503
Symptoms, n (%)				
	Asymptomatic	24 (2.9)	4 (3.6)	0.765
	Fever	667 (81.1)	77 (68.8)	0.002
	Dry cough	500 (60.8)	71 (63.4)	0.601
	Sputum production	182 (22.1)	18 (16.1)	0.142
	Fatigue	273 (33.2)	24 (21.4)	0.012
	Myalgia	66 (8.0)	6 (5.4)	0.32
	Dyspnoea/pant	292 (35.5)	10 (8.9)	0
	Vomiting/diarrhea/nausea	43 (5.2)	6 (5.4)	0.955
	Abdominal pains/diarrhea	116 (14.1)	15 (13.4)	0.837
	Sore throat/throat discomfort	40 (4.9)	5 (4.5)	0.852
	Headache/dizziness	47 (5.7)	7 (6.3)	0.821
	Chest distress	196 (23.8)	13 (11.6)	0.006
Coexisting comorbidities, n (%)				
	Hypertension	164 (20.0)	11 (9.8)	0.01
	Coronary heart diseases	72 (8.8)	4 (3.6)	0.06
	Cancer	25 (3.0)	2 (1.8)	0.762
	Pulmonary diseases	48 (5.8)	5 (4.5)	0.555
	Cerebrovascular diseases	26 (3.2)	0 (0.0)	0.062
	Diabetes	246 (29.9)	22 (19.6)	0.024
Treatment, n (%)				
	Antiviral therapy	750 (91.2)	100 (89.3)	0.497
	Antibiotic therapy	621 (75.6)	86 (76.8)	0.774

**Table S1. Continued**			
Invasive mechanical ventilation	85 (10.3)	6 (5.4)	0.095
Glucocorticoid therapy	394 (47.9)	39 (34.8)	0.0009
Hypoglycemic therapy, n/N (%)			
Only insulin therapy	73/168 (43.5)	3/12 (25.0)	0.211
Insulin and OAH therapy	63/168 (37.5)	5/12 (41.7)	0.767
Only OAH therapy	32/168 (19.1)	4/12 (33.3)	0.262
Death, n (%)	73 (8.9)	8 (7.1)	0.54

ALT alanine transaminase, APTT activated partial thromboplastin time, AST aspartate transaminase, BNP brain natriuretic peptide, CK-MB creatine phosphokinase-MB, COVID-19 the Coronavirus disease 2019, cTnI cardiac troponin I, DBP diastolic pressure, DM diabetes mellitus, FPG fasting plasma glucose, HDL-c high-density lipoprotein cholesterol, INR international normalized ratio, LDL-c low-density lipoprotein cholesterol, OAH oral anti-hyperglycemia, PT prothrombin time, RR respiratory rate, SBP systolic pressure, TC total cholesterol, TG triglycerides, TT thrombin time.

Figure S1. The incidence of severe complications of COVID-19 patients with and without DM.

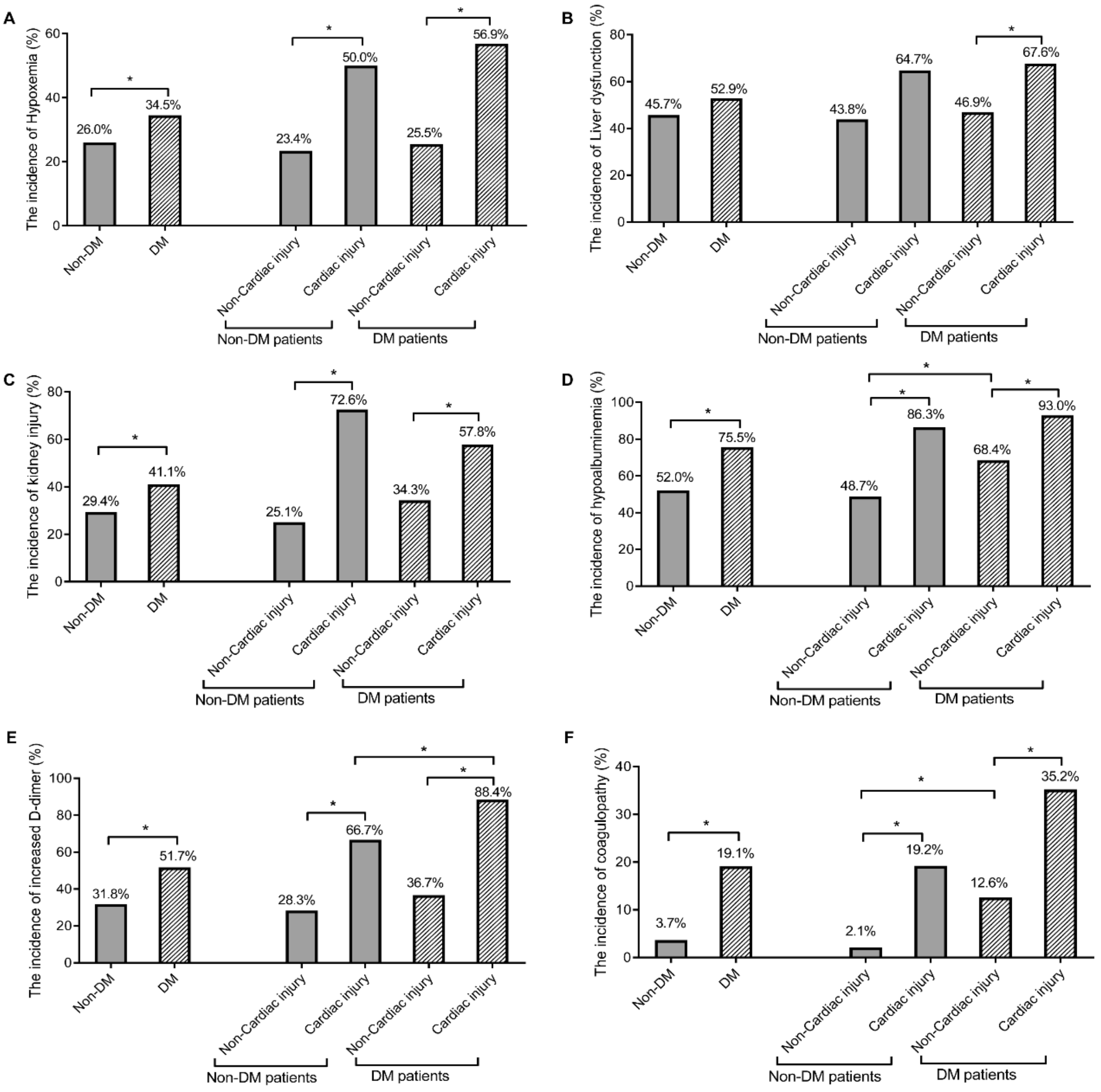

DM patients with cardiac injury show a relatively higher incidence of Hypoexmia (A), Liver dysfunction (B), kidney injury (C), hypoalbuminemia (D), increased D-dimer (E), and coagulopathy (F). *present $p<0.05$. DM diabetes mellitus.

Figure S2. The percent of immunocyte subsets in COVID-19 patients with cardiac injury grouped by DM and non-DM.

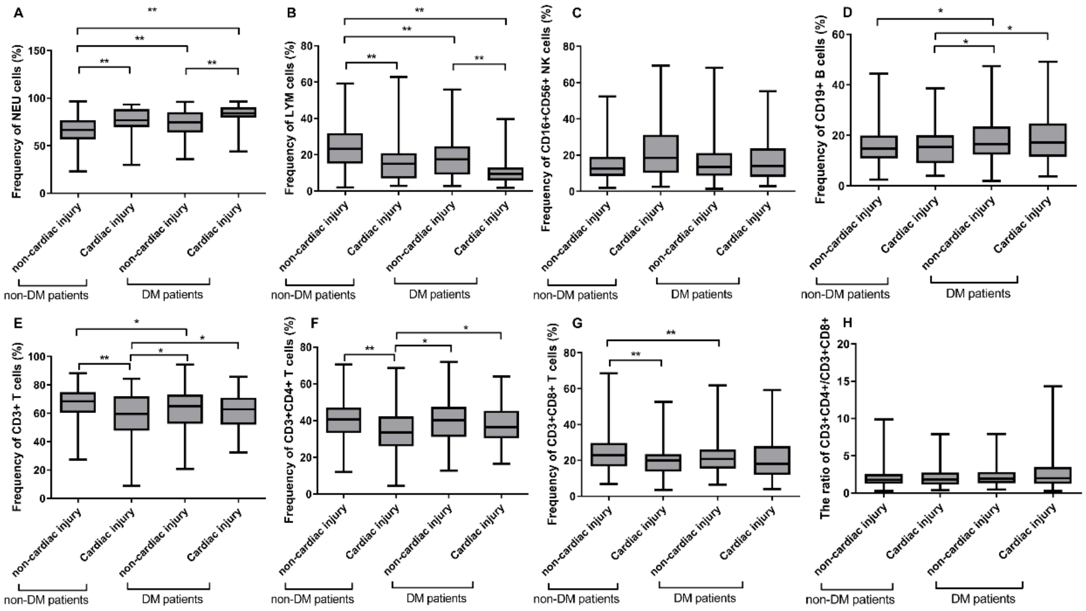

*present $p<0.05$, **present $p<0.01$. DM diabetes mellitus, NEU neutrophils, LYM lymphocytes, NK cells natural killer cells.

Figure S3. ROC analysis of immune-inflammatory parameters for in-hospital mortality of all COVID-19 patients.

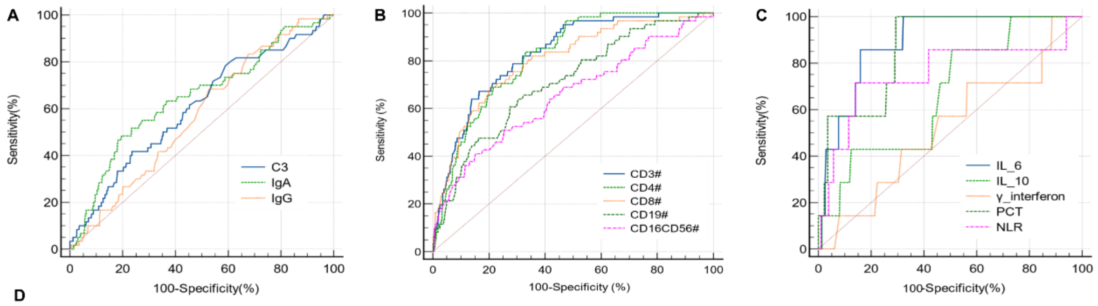

D

Variables	Descriptions	AUC	95% CI	Cut-off value	Sensitivity (%)	Specificity (%)	*P* value
C3	C3 complement, g/L	0.606	0.570-0.641	≤1.06	78.33	41.03	0.0062
IgA	Immunoglobulin A, g/L	0.647	0.612-0.681	＞3.18	48.33	80.34	0.0002
IgG	Immunoglobulin G, g/L	0.572	0.536-0.608	＞10.5	83.33	32.28	0.0387
CD3#	Number of $CD3^+$ T cells, cells/ul	0.830	0.802-0.856	≤333.00	67.21	83.52	＜0.001
CD4#	Number of $CD3^+CD4^+$ T cells, cells/ul	0.819	0.790-0.846	≤288.00	83.61	67.04	＜0.001
CD8#	Number of $CD3^+CD8^+$ T cells, cells/ul	0.801	0.771-0.828	≤114.00	68.85	79.61	＜0.001
CD19#	Number of $CD19^+$ B cells, cells/ul	0.709	0.676-0.741	≤102.00	63.93	69.55	＜0.001
CD16CD56#	Number of NK cells, cells/ul	0.653	0.619-0.687	≤59.00	40.98	84.92	＜0.001
IL-6	Interleukin 6, pg/mL	0.873	0.842-0.900	＞25.68	75.00	87.24	＜0.001
IL-10	Interleukin 10, pg/mL	0.670	0.619-0.718	＞5.71	85.71	48.61	0.0983
γ-interferon	γ-interferon, pg/mL	0.531	0.478-0.583	≤3.58	71.43	44.44	0.7995
PCT	Procalcitonin, ng/mL	0.825	0.794-0.852	＞0.078	89.71	62.28	＜0.001
NLR	Neutrophil to Lymphocyte Ratio	0.846	0.819-0.870	＞7.525	75.34	81.88	＜0.001

(A) and (B) present innate immune and lymphocyte subset absolute counts for mortality, respectively, while (C) shows the performance of inflammatory-related biomarkers on death. The optimal cut-off value of immune-inflammatory parameters in distinguishing fatal cases from survivors (D). AUC area under curve, C3 complement 3, CI confidence interval, IgA immunoglobulin A, IgG immunoglobulin G, IL-6 interleukin-6, IL-10 interleukin-10, NK cells natural killer cells, NLR neutrophils to lymphocytes ratio, PCT procalcitonin.
